# BEX3 contributes to cisplatin chemoresistance in nasopharyngeal carcinoma

**DOI:** 10.1002/cam4.982

**Published:** 2017-01-13

**Authors:** Wei Gao, John Zeng‐Hong Li, Si‐Qi Chen, Chiao‐Yun Chu, Jimmy Yu‐Wai Chan, Thian‐Sze Wong

**Affiliations:** ^1^ Department of SurgeryThe University of Hong KongHong Kong SARChina; ^2^ Department of OtolaryngologyThe First People's Hospital of FoshanGuangdong ProvinceChina

**Keywords:** Acquired resistance, BEX3, cisplatin, nasopharyngeal carcinoma, OCT4

## Abstract

Nasopharyngeal carcinoma (NPC) can develop cisplatin‐resistant phenotype. Research has revealed that enriched in cancer stem cell population is involved in developing cisplatin‐resistant phenotype. CD271 is a candidate stem cell maker in head and neck cancers. The CD receptor does not possess any enzymatic property. Signal transduction function of CD271 is mediated by the cellular receptor‐associated protein. Our data showed that Brain‐expressed X‐linked 3 (BEX3), a CD271 receptor‐associated protein, was overexpressed in NPC. BEX3 overexpression was a unique event in cancer developed in the head and neck regions, especially NPC. BEX3 expression was inducible by cisplatin in NPC. In cisplatin‐resistant NPC xenograft, treatment with nontoxic level of cisplatin led to a remarkable increase in BEX3 level. High BEX3 expression was accompanied with high octamer‐binding transcription factor 4 (OCT4) expression in cisplatin‐resistant NPC. To confirm the inducing role of BEX3 on OCT4 expression, we knockdown BEX3 using siRNA and compared the expression of OCT4 with mock transfectants. Suppressing BEX3 transcripts led to a significant reduction in OCT4. In addition, targeting BEX3 using shRNA could increase the sensitivity of NPC cells to cisplatin. In summary, our results indicated a unique functional role of BEX3 in mediating the sensitivity of NPC cells to cisplatin. Targeting or blocking BEX3 activity might be useful in reversing the cisplatin‐resistant phenotype in NPC.

## Introduction

Nasopharyngeal carcinoma (NPC) is one of the most common head and neck cancers in southern China and Southeast Asia 1, 2. Guangdong province in China and Hong Kong have the highest incidence rate around the world with incidences ranging from 20 to 22 per 100,000 men and 8–10 per 100,000 females 3. The mainstay treatment for early NPC patients is radiation therapy 4, 5. For locoregionally advanced NPC, using induction chemotherapy and concurrent chemo‐radiation demonstrated survival advantage 6, 7. Of which, therapy using platinum‐based chemotherapeutic agents such as cisplatin shows higher response rate in comparison with the nonplatinum therapy 6. Thus, the resistance of NPC to cisplatin will affect the treatment efficacy and prognosis of NPC patients.

Existence of tumorigenic cancer cell with stem cell property impedes cancer treatment efficacy. These cancer stem cells (CSC) had distinct phenotypic features with close similarities to normal stem cells. They have adaptive advantage to survive in anticancer therapy 8. In comparison to the tumor bulk, the subpopulation of CSC is heterogeneous with respect to the responsiveness of chemotherapy 8. Increasing evidence suggests that the resistance nature of CSC is a contributory cause leading to cancer recurrence 9. The resting stem cell phenotype makes them less responsive to drugs which target preferentially to actively proliferating cancer cells 10. In addition, CSC had proficient DNA repair capacity with slower cell cycle making them less responsive to chemotherapeutic agents which target actively proliferating cells 11.

CD271 antigen is a neurotrophin receptor expressed on the epithelial stem cell surface. Recent data suggest that CD271 is a functional receptor expressed on the head and neck CSC 12. High CD271 expression is found in the oral cancer with less differentiated phenotype 13. CD271‐positive hypopharyngeal cancer cells had higher tumorigenicity than the CD271‐negative counterparts in vivo and had higher resistance against chemotherapy 14. Increased CD271 expression is associated with poor prognosis of patients with oral and hypopharyngeal carcinoma 12, 13, 14. At present, whether CD271 has any functional and clinical impact on NPC remains to be elusive. CD271 itself did not possess any enzymatic activity. Signal transduction function of CD271 is mediated by the cellular receptor‐associated protein.

Octamer‐binding transcription factor 4 (OCT4) is a POU‐domain transcription factor. It is a well‐known stem cell marker and its functional role in mediating chemoresistance has been reported in a wide variety of human cancers 15, 16, 17. Chemoresistant hepatocellular carcinoma (HCC) cell lines with CSC characteristics showed a dramatically upregulated expression level of OCT4 15. Overexpression of OCT4 enhanced the resistance of HCC cells to chemotherapeutic drugs by activating AKT signaling pathways. Elevated OCT4 expression was observed in oxaliplatin‐resistant colorectal cancer (CRC) cell lines with CSC properties 16. OCT4 could activate Signal Transducer and Activator of Transcription 3 (STAT3) pathway, leading to an increase in antiapoptotic property of chemoresistant CRC cells. In head and neck cancer, forced OCT4 expression promoted the conversion of differentiated cells into CSC and conferred resistance to cisplatin 17.

To explore the potential clinical implication of CD271 in NPC, we examined the NPC transcriptome data in the public repository. In the pretreatment NPC, CD271 is not highly expressed. Our data revealed that the CD271‐associated protein, Brain‐expressed X‐linked 3 (BEX3), showed remarkable upregulation in the primary NPC. BEX3 expression was inducible in response to cisplatin treatment. Furthermore, significant upregulation of BEX3 was observed in cisplatin‐resistant NPC cells. Thus, we proposed that BEX3 overexpression is important for the NPC cell to enrich the stemness features and acquire cisplatin‐resistant phenotype, which thereby allows the cancer cells to withstand the stressful environment created by the genotoxic chemotherapeutic agents.

## Materials and Methods

### Microarray and in silico analysis

Human cancer microarray meta‐analysis was performed using CancerMA 18. The database contains 80 microarray datasets using Affymetrix HG‐U133 Plus 2 array from ArrayExpress or the GEO repository. These datasets cover 13 cancer types including adrenal, brain, breast, colorectal, head and neck, leukemia, lung, lymphoma, ovarian, pancreatic, prostate, renal, and thyroid. Individual datasets were normalized and the expression values were computed by the ‘affy’ R package from Bioconductor. Subsequently, the differentially expressed genes were computed by the ‘Limma’ R package from Bioconductor and *P*‐values were adjusted by the Benjamini and Hochberg's method. Then, a meta‐analysis was conducted to combine the results of individual datasets for each cancer type and a meta‐ *P*‐value and a meta‐log2‐fold change value were calculated. A |meta‐log2‐fold change| >1 or a confidence interval that does not span 0, and a meta‐ *P*‐value <0.05 were considered as significant. Circos plots and forest plots were employed to visualize the analysis results. Oncomine Cancer Microarray database was used to analyze the expression of BEX3 in NPC tissues and normal tissue counterparts 19. Microarray dataset (GSE12452) containing expression data of 31 NPC tissues and 10 normal controls were obtained from GEO. The microarray data were normalized and analyzed using Gene Expression Commons 20.

### Cell cultures and chemicals

NPC cell lines HONE1 and HK1 were maintained in RPMI‐1640 medium supplemented with 10% fetal bovine serum, 200 Unit/mL penicillin G sodium, 200 *μ*g/mL streptomycin sulfate, and 0.5 *μ*g/mL amphotericin B. HONE1 was established from a poorly differentiated NPC. HK1 was derived from a well‐differentiated NPC 21, 22. Unless specified otherwise, all the chemicals were obtained from Sigma.

### Immunocytochemistry

NPC cells were fixed with 4% paraformaldehyde and permeabilized using 0.25% Triton X‐100 in PBS. Immunoreactions were performed using anti‐BEX3 (R&D Systems, Minneapolis, MN), anti‐OCT4 (Santa Cruz, Dallas, TX) and anti‐CD271 (Santa Cruz) antibodies. CF488‐conjugated secondary antibodies (Biotium, Fremont, CA) was used to develop the signals. Blue‐fluorescent 4’,6‐Diamidino‐2‐Phenylindole (DAPI, Invitrogen) was used to stain the nucleus. F‐actin was stained by red‐fluorescent Alexa Fluor^®^ 635 phalloidin (Invitrogen, Grand Island, NY). Images of the immunostaining were captured using fluorescent microscope (Nikon, Tokyo, Japan).

### Western blot analysis

Total protein was extracted by RIPA buffer and protein concentration was determined by BCA Protein Assay Kit (Pierce Biotechnology, Waltham, MA). Protein was separated by SDS‐PAGE and transferred to polyvinylidene difluoride (PVDF) membrane (Millipore, Billerica, MA) using semidry method. Then, protein was hybridized to anti‐BEX3 (R&D Systems), anti‐OCT4 (Santa Cruz) and anti‐CD271 (Santa Cruz) antibodies, followed by incubation with horse radish peroxidase (HRP) conjugated goat anti‐mouse IgG (Invitrogen). ECL Plus Western Blotting Detection Reagents (Amersham Biosciences, Pittburgh, PA) was used to develop hybridization signals.

### Real‐time quantitative polymerase chain reaction

Total RNA was reverse transcribed using High Capacity cDNA Reverse Transcription Kit (ABI). Sequences of Real‐time quantitative polymerase chain reaction (QPCR) primers were as follows: BEX3 (forward primer: 5′‐CTTCGGTGCAGTCGTCACT‐3′; reverse primer: 5′‐ACACTTAGCCTCGCAGACCT‐3′; Universal ProbeLibrary probe number 24); OCT4 (forward primer: 5′‐CTTCGCAAGCCCTCATTTC‐3′; reverse primer: 5′‐GAGAAGGCGAAATCCGAAG‐3′; probe number 60). Reactions were performed at 95°C for 10 min followed by 45 cycles of 95°C for 15 sec and 60°C for 1 min. Expression levels were evaluated using the comparative threshold cycle methods. All the reactions were performed using FastStart Universal Probe Master (Roche Applied Science) on LightCycler^®^ 480 (Roche Applied Science). PCR quantification was calculated using 2^−ΔΔct^ method against the glyceraldehyde‐3‐phosphate dehydrogenase (GAPDH) for normalization.

### Development of cisplatin‐resistant HONE1

The cisplatin‐resistant HONE1 cell line was developed by chronic treatment of cisplatin. HONE1 was exposed to cisplatin for 3 days, followed by growth recovery in drug‐free medium. The concentration of cisplatin was increased in the subsequent cycle and the procedure was repeated until resistance was achieved in HONE1. After 21 cycles, the level of cisplatin resistance was determined by in vitro toxicity test.

### In vitro toxicity test

In vitro toxicity assay was used to confirm the resistance level of cisplatin‐resistant HONE1 cell line. Relative cell viability of cells after cisplatin treatment was determined by in vitro Toxicology Assay Kit Sulforhodamine B assay (TOX6 from Sigma‐Aldrich, St. Louis, MO) according to manufacturer's protocol. The percentage of viability of cells was calculated by number of cisplatin‐treated viable cells / number of viable cells of untreated control.

### Colony formation assay

Cells were seeded in a six‐well plate with 600 cells in each well. After 24 h, cells were treated by cisplatin for 72 h. After incubation for 14 days, cells were fixed with 70% ethanol and stained with 0.5% crystal violet. The numbers of colonies with more than 50 cells were counted.

### Animals

A total of 2 × 10^6^ parental HONE1 or cisplatin‐resistant HONE1 cells were injected subcutaneously into the right flank of athymic nu/nu mice (5 weeks old, weight range: 18–22 g). Tumor growth was measured daily with calipers in two dimensions. Tumor volume was calculated using the formula: Volume (in mm^3^) = (L × W^2^)/2, where L is the length in millimeters and W is the width in millimeters. When the tumor volume reached 100 mm^3^, the mice with xenograft derived from parental HONE1 or cisplatin‐resistant HONE1 were treated by cisplatin. There were five mice in each group. Cisplatin (2.5 mg/kg body weight) was administrated by intraperitoneal injection twice per week. At the end point, mice were sacrificed with an excessive dosage of pentobarbital and the subcutaneous tumors were removed.

### Immunohistochemistry

Mice xenograft were embedded into paraffin blocks and cut into 4‐*μ*m sections. The sections were dewaxed with xylene and rehydrated. Antigen retrieval was achieved by microwaving in 10 mmol/L sodium citrate buffer (pH 6.0) for 15 min. To block the endogenous peroxidase activity, the sections was incubated in 3% hydrogen peroxide for 10 minutes. Sections were stained with anti‐BEX3 antibodies (R&D Systems) at room temperature for 1 h. EnVision+ System, HRP (DAKO) was used for visualization of immunoreaction. Then, sections were counterstained with Mayer's hematoxylin, dehydrated, and photographed under light microscope.

### SiRNA transfection

BEX3 siRNA, OCT4 siRNA, and negative control siRNA were obtained from Qiagen. HiPerFect reagent (QIAGEN, Valencia, CA) was used to carry out the transfection reactions according to the manufacturer's protocol. After 72 h, cells were collected and the efficiency of BEX3 silencing was determined by QPCR and Immunocytochemistry.

### Lentivirus vector construction, lentivirus production, and infection

The oligonucleotides encoding a shRNA specific for BEX3 were cloned into EcoRI/BamHI sites of pGreenPuro vector (System Bioscience). The sense oligonucleotide is: 5’‐ GATCCGGGAGCTGCAGTTGAGGAATTCTTCCTGTCAGAAATTCCTCAACTGCAGCTCCCTTTTTG‐3’ and the antisense: 5’‐AATTCAAAAAGGGAGCTGCAGTTGAGGAATTTCTGACAGGAAGAATTCCTCAACTGCAGCTCCCG‐3’. Construct sequences were verified by direct sequencing. Virus packaging was performed by transient transfection into 293T cells using Lipofectamine^®^ 2000 transfection reagent (Invitrogen). HONE1 cells were transduced with medium containing lentivirus.

### Real‐time cell kinetic analysis

The xCELLigence RTCA (ACEA Biosciences) was used to record the real‐time changes of cell proliferation propensity of the NPC cells. Data analysis was performed using RTCA Control Unit and the preinstalled RTCA software. Cells were seeded directly onto E‐plate 16. Changes in baseline impedance resulting from cell number increase were recorded by the microelectrodes. Proportional changes in impedance were expressed in cell index (CI).

### Statistical analysis

Statistical analysis was performed using SPSS software version 20.0. Student *t*‐test was used to compare difference between groups. All the tests were two‐sided. *P* < 0.05 was considered as statistically significant.

## Results

### BEX3 was upregulated in head and neck cancers and NPC

**Figure 1:**
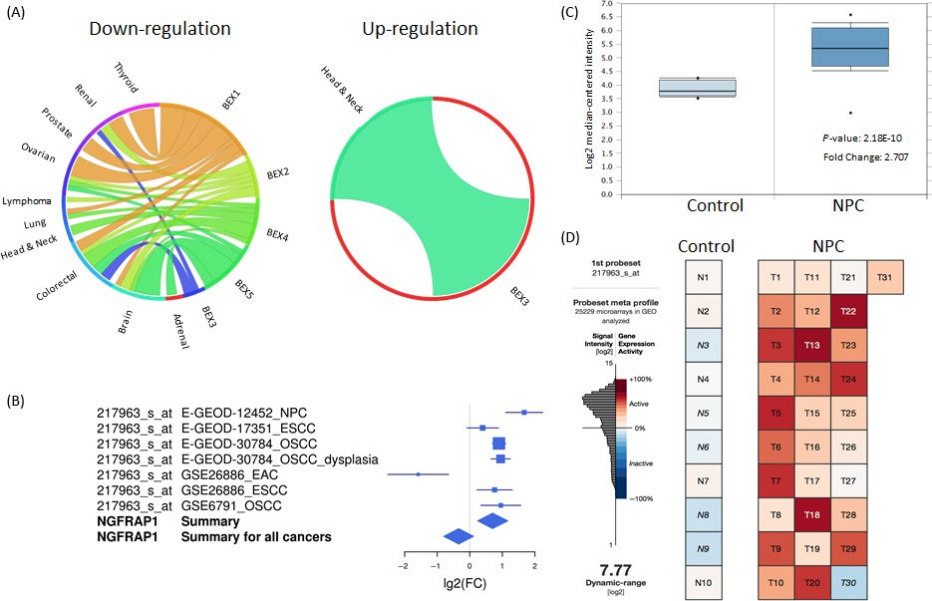
BEX3 was upregulated in head and neck cancers and NPC. (A) Circos plots showing meta‐analysis of BEX family members in human malignancies using CancerMA. (B) Forest plot showing BEX3 expression in head and neck cancers. NGFRAP1: BEX3. (C) Expression level of BEX3 in NPC and normal tissues in GSE12452 from Oncomine. (D) Expression level of BEX3 in NPC and normal tissues in GSE12452 from Gene Expression Commons. BEX3, Brain‐expressed X‐linked 3; NPC, Nasopharyngeal carcinoma.

To explore the differential expression patterns of BEX family members in human malignancies, we performed cancer microarray data meta‐analysis using CancerMA. Expression of BEX1, BEX2, BEX4, and BEX5 were found to be reduced in different human cancers (Fig. 1A). Among the 5 BEX members, BEX3 was the one and only one found to be significantly upregulated in head and neck cancers (Fig. 1A). Significant overexpression of BEX3 is observed in NPC, esophageal squamous cell carcinoma (ESCC), oral dysplasia, and oral squamous cell carcinoma (OSCC) (Fig. 1B). In comparison with all the head and neck datasets, BEX3 in NPC showed the highest fold‐increase in comparison with the normal tissues (Fig. 1B). To confirm our findings, we explored BEX3 expression level from NPC dataset in Oncomine. Marked overexpression of BEX3 was observed in the laser microdissected epithelial carcinoma cells from the NPC tissues in GSE12452 (fold change = 2.707, *P* = 2.18E^−10^; Fig. 1C). Analysis of GSE12452 by Gene Expression Commons also confirmed the overexpression of BEX3 in NPC tissues (Fig. 1D).

### Cisplatin treatment induced the expression of BEX3

**Figure 2:**
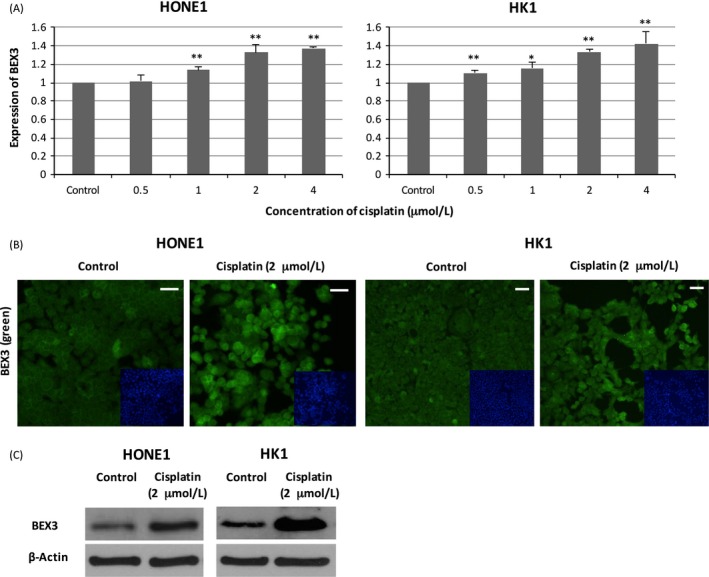
Cisplatin treatment induced the expression of BEX3. (A) QPCR analysis of mRNA expression level of BEX3 in HONE1 and HK1 cells treated with cisplatin. (B) ICC analysis of protein expression level of BEX3 in NPC cells exposed to cisplatin. Blue‐fluorescent DAPI was used to stain the nucleus. (C) Western blot analysis of protein expression level of BEX3 in NPC cells exposed to cisplatin. Error bar indicate SD,* n* = 3, *P < 0.05, **P < 0.01. Scale bar = 50 *μ*m. BEX3, Brain‐expressed X‐linked 3; ICC, Immunocytochemistry; NPC, Nasopharyngeal carcinoma; QPCR, Real‐time quantitative polymerase chain reaction.

To investigate the potential role of BEX3 in mediating cisplatin resistance, we investigated whether the expression of BEX3 was responsive to cisplatin. Cisplatin treatment at low concentration induced BEX3 mRNA expression in a dose‐dependent manner in both HONE1 and HK1 cells (Fig. 2A). Results from Immunocytochemistry (ICC) and western blot showed that cisplatin treatment also enhanced the protein expression level of BEX3 (Fig. 2B and C).

### Cisplatin‐resistant HONE1 cells exhibited higher expression of BEX3, OCT4, and CD271

**Figure 3:**
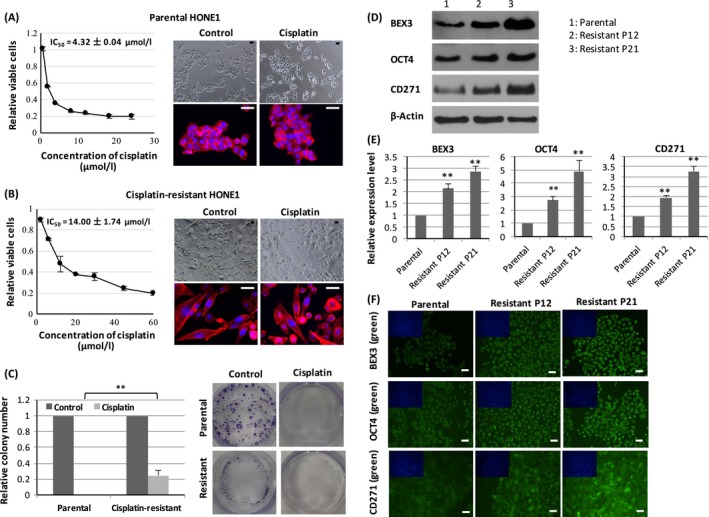
Cisplatin‐resistant HONE1 cells exhibited higher expression of BEX3, OCT4 and CD271. (A, B) In vitro toxicity assay of cisplatin in parental and cisplatin‐resistant HONE1. The IC_50_ value was calculated from the dose–response curve. Morphology of parental and cisplatin‐resistant HONE1 was shown by staining of F‐actin using red‐fluorescent phalloidin. (C) Colony formation assay of parental and cisplatin‐resistant HONE1 exposed to cisplatin. (D) Western blot analysis of protein expression level of BEX3, OCT4 and CD271 in parental and cisplatin‐resistant HONE1. (E, F) QPCR and ICC analysis of mRNA and protein expression level of BEX3, OCT4 and CD271 in parental and cisplatin‐resistant HONE1. P12, passage 12; P21, passage 21. Blue‐fluorescent DAPI was used to stain the nucleus. Error bar indicate SD,* n* = 3, **P < 0.01. Scale bar = 50 *μ*m. BEX3, Brain‐expressed X‐linked 3; ICC, Immunocytochemistry; QPCR, Real‐time quantitative polymerase chain reaction.

To explore the functional implications of BEX3 in cisplatin resistance, we generated cisplatin‐resistant HONE1 by chronic treatment with increasing doses of cisplatin. In vitro toxicity assay showed that the IC_50_ value was 3.2‐fold higher in cisplatin‐resistant HONE1 (14.00 ± 1.74 *μ*mol/L) than that of parental HONE1 (4.32 ± 0.04 *μ*mol/L, *P* < 0.01; Fig. 3A and B). The cisplatin‐resistant HONE1 cells exhibited a spindle‐like morphology, indicating a more aggressive phenotype (Fig. 3A and B). In colony formation assay, no colony was formed in parental HONE1 exposed to 10 *μ*mol/L cisplatin (Fig. 3C). In contrast, a significantly higher number of colony was observed in cisplatin‐resistant HONE1 after cisplatin treatment compared to parental HONE1 (Fig. 3C). Given that CSC contributed to resistance to cisplatin treatment, we explored the expression level of OCT4 (an embryonic stem cell marker) and CD271 (a CSC marker) in cisplatin‐resistant HONE1. The expression levels of BEX3, OCT4 and CD271 were compared in various passages (passage 12 and 21) of cisplatin‐resistant HONE1. Results from western blot revealed that the protein expression levels of BEX3, OCT4 and CD271 were increased in cisplatin‐resistant HONE1 (Fig. 3D). QPCR and ICC showed that the mRNA and protein levels of BEX3, OCT4, and CD271 were elevated in parallel with increasing passage number (Fig. 3E and F).

### Cisplatin treatment induced the expression of BEX3 in mouse xenograft model

**Figure 4:**
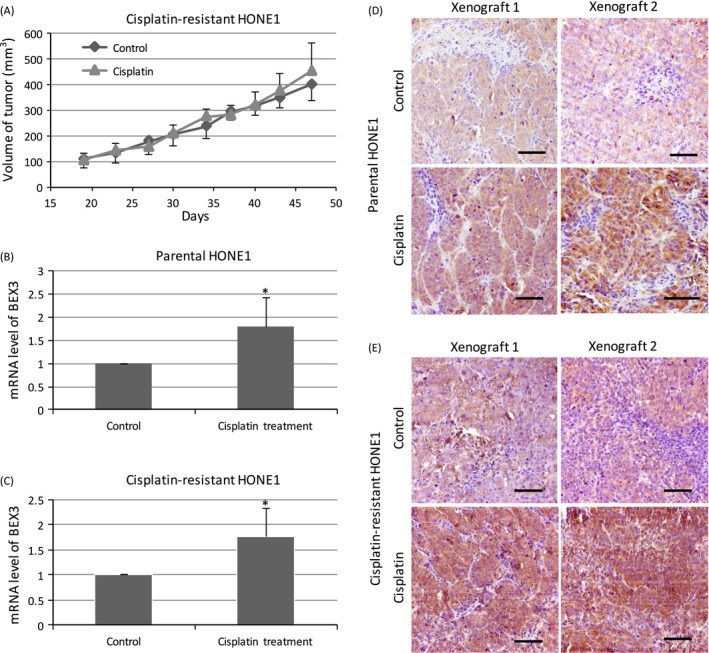
Cisplatin treatment induced the expression of BEX3 in mouse xenograft model. (A) Growth curve of xenograft derived from cisplatin‐resistant HONE1 upon cisplatin treatment. There were five mice in each group, Error bar indicate SE. (B, C) QPCR analysis of mRNA expression level of BEX3 in xenograft derived from parental HONE1 or cisplatin‐resistant HONE1 upon cisplatin treatment. Error bar indicate SD,* n* = 5, *P < 0.05. (D, E) IHC analysis of protein expression level of BEX3 in xenograft derived from parental HONE1 or cisplatin‐resistant HONE1 under cisplatin treatment. Scale bar = 50 *μ*m. BEX3, Brain‐expressed X‐linked 3; IHC, Immunohistochemistry; QPCR, Real‐time quantitative polymerase chain reaction.

To further study the BEX3‐inducing effects of cisplatin in vivo, we generated NPC xenograft using parental HONE1 or cisplatin‐resistant HONE1 and examined the changes of BEX3 after exposure to cisplatin. For xenograft derived from cisplatin‐resistant HONE1, administration of cisplatin at the concentration of 2.5 mg/kg body weight for 28 days did not have significant impact on xenograft growth, implicating the resistance to cisplatin treatment (Fig. 4a). QPCR and Immunohistochemistry (IHC) showed that cisplatin treatment enhanced the mRNA (Fig. 4B and C) and protein (Fig. 4D and E) expression levels of BEX3 in xenograft derived from both parental HONE1 and cisplatin‐resistant HONE1.

### BEX3 regulated the expression of OCT4

**Figure 5:**
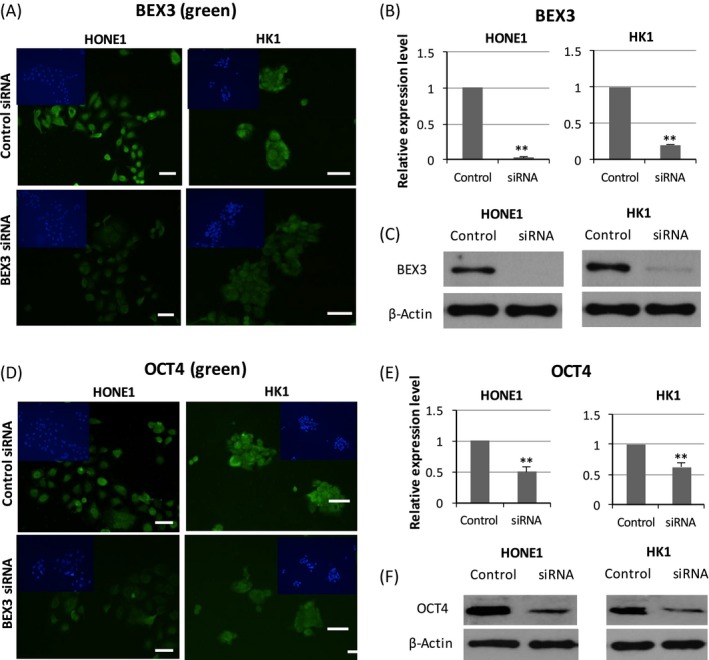
BEX3 regulated the expression of OCT4. (A, B) ICC and QPCR analysis of protein and mRNA expression level of BEX3 in HONE1 and HK1 cells transfected with BEX3 siRNA. (C) Western blot analysis of protein expression level of BEX3 in HONE1 and HK1 cells transfected with BEX3 siRNA. (D, E) ICC and QPCR analysis of protein and mRNA expression level of OCT4 in NPC cells transfected with BEX3 siRNA. Blue‐fluorescent DAPI was used to stain the nucleus. (F) Western blot analysis of protein expression level of OCT4 in HONE1 and HK1 cells transfected with BEX3 siRNA. Error bar indicate SD,* n* = 3, **P < 0.01. Scale bar = 50 *μ*m. BEX3, Brain‐expressed X‐linked 3; ICC, Immunocytochemistry; DAPI, 6‐Diamidino‐2‐Phenylindole; NPC, Nasopharyngeal carcinoma; QPCR, Real‐time quantitative polymerase chain reaction.

Given that high BEX3 expression was accompanied with high OCT4 expression in cisplatin‐resistant HONE1 cells, we investigated the potential role of BEX3 in modulating OCT4. Results from ICC, western blot and QPCR revealed that transfection of HONE1 and HK1 cells with 15 nM BEX3 siRNA successfully reduced the protein (Fig. 5A and C) and mRNA (Fig. 5B) levels of BEX3. Silence of BEX3 resulted in a significant decrease in protein (Fig. 5D and F) and mRNA (Fig. 5E) levels of OCT4.

### BEX3 knockdown sensitized NPC to cisplatin

**Figure 6:**
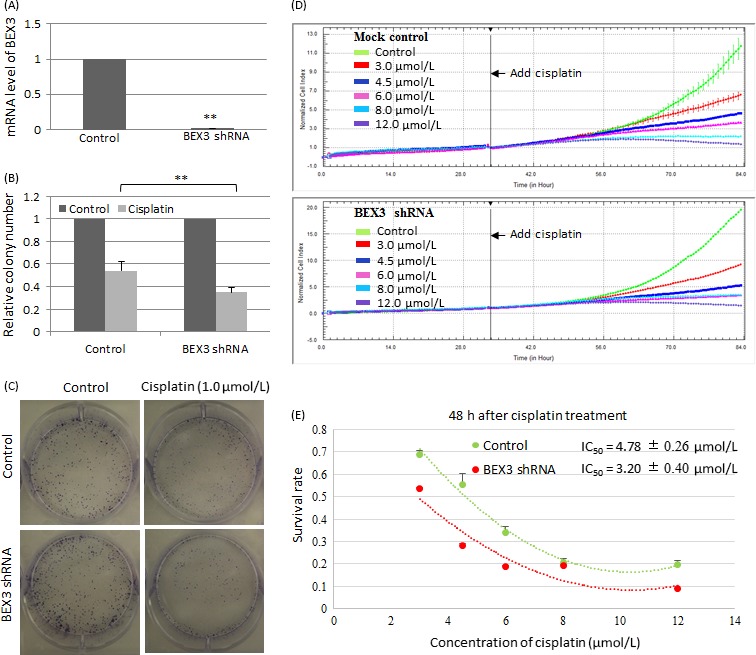
BEX3 knockdown sensitized NPC to cisplatin. (A) QPCR analysis of mRNA expression level of BEX3 in HONE1 with BEX3‐shRNA. (B, C) Colony formation of BEX3‐knockdown HONE1 cells after exposure to 1.0 *μ*mol/L cisplatin. (D, E) Real‐time cell kinetic analysis of cisplatin cytotoxicity using xCELLigence RTCA. Cisplatin was added to BEX3‐knockdown HONE1 cells and cell index was continuously monitored. The IC_50_ value was calculated from the dose–response curve after cisplatin treatment for 48 h. Error bar indicate SD,* n* = 3, **P < 0.01. BEX3, Brain‐expressed X‐linked 3; ICC, Immunocytochemistry; NPC, Nasopharyngeal carcinoma; QPCR, Real‐time quantitative polymerase chain reaction.

HONE1 cells containing BEX3‐shRNA was generated by lentivirus transduction. Downregulation of BEX3 mRNA level was observed in HONE1 with BEX3‐shRNA (Fig. 6A). Results from colony formation assay showed that less colonies were observed in BEX3‐knockdown cells after exposure to 1.0 *μ*mol/L cisplatin (Fig. 6B and C). The IC_50_ value of cisplatin was evaluated by the xCELLigence RTCA. Cells were seeded on E‐plate and incubated for 34 h before cisplatin was added to cells. After cisplatin treatment for 48 hours, the IC_50_ value was calculated. The IC_50_ value was significantly lower in HONE1 cells containing BEX3‐shRNA (3.20 ± 0.40 ***μ***mol/L) compared to that of mock control (4.78 ± 0.26 ***μ***mol/L, *P* < 0.05; Fig. 6D and E).

### BEX3 knockdown sensitized cisplatin‐resistant NPC to cisplatin

**Figure 7:**
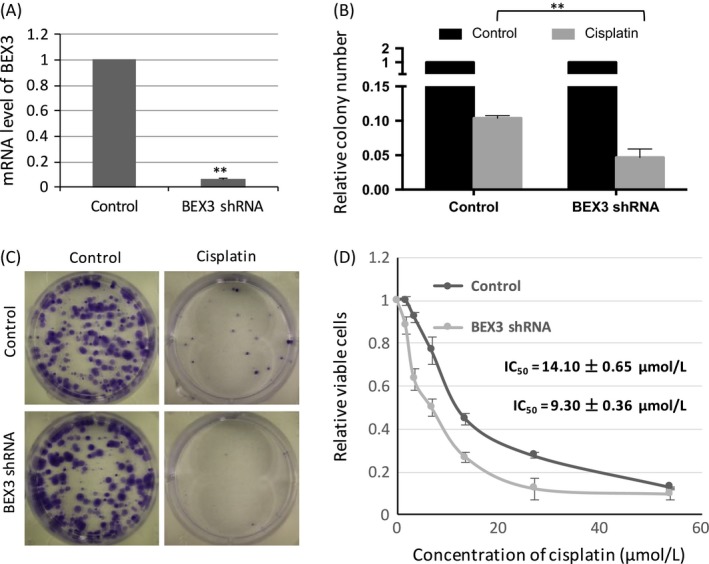
BEX3 knockdown sensitized cisplatin‐resistant NPC to cisplatin. (A) QPCR analysis of mRNA expression level of BEX3 in cisplatin‐resistant HONE1 with BEX3‐shRNA. (B, C) Colony formation of cisplatin‐resistant HONE1 cells with BEX3‐shRNA upon cisplatin treatment. (D) Dose–response curve of cisplatin‐resistant HONE1 with BEX3‐shRNA and mock control cells under cisplatin treatment. The IC_50_ value of cisplatin was calculated from dose–response curve. Error bar indicate SD,* n* = 3, **P < 0.01. BEX3, Brain‐expressed X‐linked 3; ICC, Immunocytochemistry; QPCR, Real‐time quantitative polymerase chain reaction.

Cisplatin‐resistant HONE1 with BEX3‐shRNA exhibited reduced mRNA expression level of BEX3 (Fig. 7A). In colony formation assay, BEX3‐knockdown cells showed less colonies upon cisplatin treatment (Fig. 7B and C). The IC_50_ value of cisplatin was determined by in vitro toxicity test. A significant lower IC_50_ value was observed in BEX3‐knockdown cells (9.30 ± 0.36 *μ*mol/L) in comparison with mock control (14.10 ± 0.65 *μ*mol/L, *P* < 0.01; Fig. 7D).

### Knockdown of OCT4 enhanced the sensitivity of NPC to cisplatin

**Figure 8:**
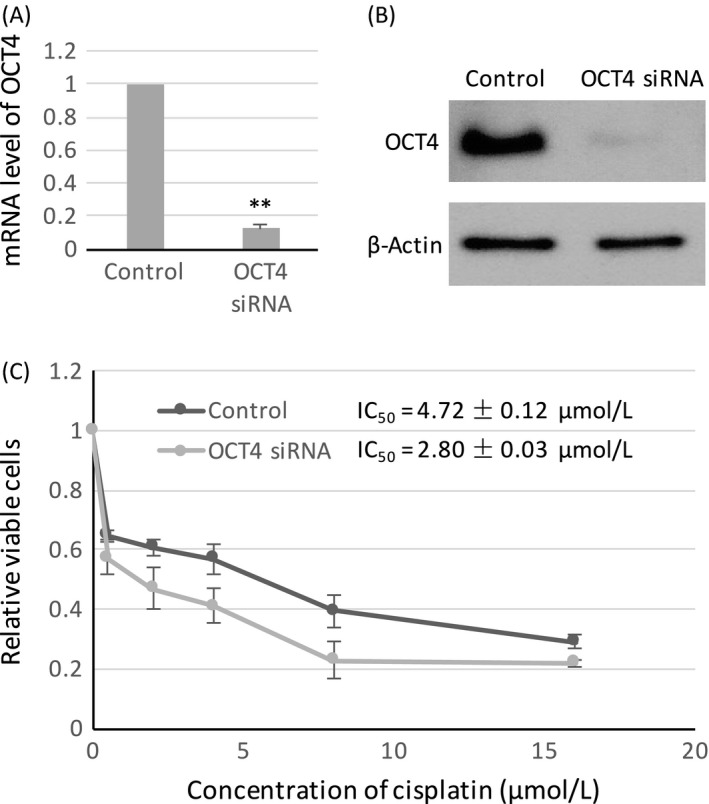
OCT4 knockdown sensitized NPC to cisplatin. (A) QPCR analysis of mRNA expression level of OCT4 in HONE1 transfected with OCT4 siRNA. (B) Western blot analysis of protein expression level of OCT4 in HONE1 transfected with OCT4 siRNA. (C) Dose–response curve of OCT4‐knockdown HONE1 and mock control cells under cisplatin treatment. The IC_50_ value of cisplatin was calculated from dose–response curve. Error bar indicate SD,* n* = 3, **P < 0.01. NPC, Nasopharyngeal carcinoma; QPCR, Real‐time quantitative polymerase chain reaction.

The mRNA (Fig. 8A) and protein (Fig. 8B) expression levels of OCT4 were significantly decreased in HONE1 cells transfected with OCT4 siRNA. Under cisplatin treatment, the IC_50_ value of OCT4‐knockdown cells (2.80 ± 0.03 *μ*mol/L) was significantly lower than mock control (4.72 ± 0.12 *μ*mol/L, *P* < 0.01; Fig. 8C).

## Discussion

NPC patients suffering from relapse to therapy or recurrent disease are resistant to the conventional therapy with radiation or chemotherapeutic drugs 23. Stimulation from the tumor microenvironment shapes the development of CSC and development of resistance against chemotherapy. Cancer cells can be induced to acquire the stem cell phenotype under chronic chemotherapeutic stress. Chemotherapeutic drugs can trigger adaptive response at multiple levels and promote cancer cells to acquire stem cell phenotype in order to potentiate survival in the stressful environment.

Cis‐diamminedichloroplatinum (II) or cisplatin is a platinum‐based genotoxic drug used most often in the treatment of NPC with satisfactory response rate 24. Cisplatin could render cell cycle arrest and induce apoptosis by forming adducts with the DNA of the cancer cells. Cisplatin‐based chemotherapy has been shown to improve the overall and progression‐free survival of NPC patients 25, 26. Nevertheless, treatment failure is not uncommon as the cancer may be inherent resistant to cisplatin or acquire resistant phenotype during the course of treatment 27. Patients typically develop cisplatin resistance within 2 years of initial treatment leading to poor prognosis. At present, there is still no effective pharmacological manipulation available to circumvent the problem.

CD271 is expressed at high level in embryonic stem cells and adult stem cells. In oral epithelia cancer, high CD271 expression is associated with poor prognosis 28. CD271 do not have any intrinsic enzymatic property. The signal transduction functions are achieved by recruiting intracellular proteins which interact with the intracellular domains of CD271 29. Hence, identifying and evaluating key receptor‐associated protein is important to understand the functional implication of CD271 in regulating therapeutic sensitivity.

In normal neuronal cells, multiple CD271 binding proteins have been identified. The interactions between different receptor‐associated protein could regulate different signal cascade and control multiple cellular processes including cell cycle, migration, invasion, and apoptosis 29. Our results showed that BEX3, the receptor‐associated protein, is significantly increased in NPC tissues. BEX3 expression is further increased in response to cisplatin treatment. The association of BEX3 in cisplatin resistance is further confirmed with the established cisplatin‐resistant cell line. Overall, the results suggested that upregulating BEX3 expression is important for the NPC cells to response to the genotoxic stress introduced by cisplatin.

The functional implications of BEX3 in cancers remained unresolved. At present, most studies on normal neural originated cells suggest that BEX3 is a proapoptotic gene 30. In normal cells, BEX3 is known as a cell death executor as it could mediate cell death in the presence of CD271 ligand 31. In cultured cortical neurons, BEX3 could induce caspase‐dependent neuronal apoptosis 32. In breast cancers, BEX3 promoted apoptosis and inhibited mouse xenograft formation 33. On the contrary, several studies showed the prosurvival role of BEX3. Silence of BEX3 reduced the survival of nerve growth factor (NGF)‐dependent neurons by inhibiting the expression of tyrosine kinase receptor A of NGF 34. In F9 teratocarcinoma cells, knockdown the expression of BEX3 suppressed cell growth 35. Thus, high expression of BEX3 in NPC with uncontrolled proliferative capability seems to be contradicting with the proposed functions in cell death. To explore expression patterns of BEX family in human malignancies, we performed microarray meta‐analysis on 13 cancer types. The results indicated that BEX3 upregulation is only limited on cancers originated in head and neck. Upregulation is not observed on other cancer types including adrenal, brain, breast colorectal, leukemia, lung, lymphoma, ovarian, pancreatic, prostate, renal, and thyroid. The data reveal that BEX3 overexpression is dependent on cell context. The confined distribution in the human cancers indicated that BEX3 is playing a unique role in the pathogenesis of cancers in head and neck regions including NPC.

As shown in Oct4‐inducible mice, continuous Oct4 expression in epithelial tissues could lead to dysplastic growth suggesting that high Oct4 may account for the cancerous changes of the epithelial layers 36. Exogenous expression of OCT4 could increase the tumor‐initiating and colonization capabilities of cancer cells 37. In noncancerous head and neck epithelia, OCT4 expression level is beyond the detection limit and could only be detected in the basal layer 38. In NPC tissue, however, OCT4 is overexpressed with high level detected at the invasive front of the cancer tissues and is associated with the poor overall survival of NPC patients 38. OCT4 expression is enriched in NPC with stem cell characteristics 39. NPC with high sphere forming ability exhibited significantly higher OCT4 expression 40.

In summary, our results indicate that BEX3 expression is linked with cisplatin sensitivity of NPC. Targeting BEX3 might be clinically useful for sensitizing NPC to cisplatin‐based chemotherapy. Further studies on the functional role of BEX3 are warranted for developing the potential therapeutic use of BEX3 as molecular target in NPC treatment.

## Conflict of Interest

The authors declare no financial support or relationship that may pose conflict of interest.
